# Conservative Treatment Including Focused Extracorporeal Shockwave Therapy as a First-Line Treatment for Isolated Peroneus Longus Tendon Tear in a Professional Football Player Shows Excellent Clinical and Radiological Outcomes: A Case Report

**DOI:** 10.3390/life16071129

**Published:** 2026-07-07

**Authors:** L. Alejandro Culebras Almeida, Adrien J.-P. Schwitzguebel

**Affiliations:** 1 Centre de Médecine du Sport et de l'Exercice, Swiss Olympic Medical Center, Hirslanden Clinique la Colline, 1206 Geneva, Switzerland; 2 AS Médecine du Sport, Hôpital de la Providence, 2000 Neuchatel, Switzerland

**Keywords:** focused shockwave therapy, peroneus longus, tendon tear, ankle injury, return-to-play, case report

## Abstract

Introduction: Isolated peroneus longus (PL) tendon tears are rare injuries and an often-overlooked cause of chronic lateral ankle pain. Evidence regarding optimal conservative management, especially in elite athletes, remains limited. Focused extracorporeal shockwave therapy (F-ESWT) has shown promising results in tendinopathies, yet its application in acute tendon tears is poorly documented. Methods: We present the case of a professional football player in his late twenties who sustained an isolated longitudinal PL tear. The player underwent a combined conservative protocol including F-ESWT, physiotherapy, and custom orthotics. Pain (Visual Analog Scale, VAS) and function (AOFAS Ankle–Hindfoot Score) were assessed at baseline, three months, and in an 18-month follow-up. MRI scans were obtained at diagnosis and after six weeks to evaluate tendon healing. Results: The conservative treatment protocol, including eight sessions of ultrasound-guided F-ESWT (2500–4000 impulses per session, 50–550 µJ/mm^2^) led to marked pain relief and functional improvement. VAS decreased from 7/10 to 1/10, while AOFAS improved from 57 to 95 points, maintained at 18 months. MRI after six weeks of treatment demonstrated decreased intratendinous hyperintensity, reduced lesion length, and re-establishment of normal tendon architecture. The player returned to training after eight weeks and full competition after 12 weeks, remaining asymptomatic thereafter. Conclusions: A multimodal treatment consisting of F-ESWT, physiotherapy with progressive loading and orthotic correction resulted in rapid and durable healing of an isolated PL tear in a professional athlete. This case supports the potential role of a multimodal conservative treatment strategy including F-ESWT as a first-line, non-invasive option for acute peroneal tendon tears.

## 1. Introduction

Ankle and foot injuries represent a substantial burden in professional football, accounting for nearly 15–20% of all sports-related musculoskeletal lesions. Most involve lateral ligament sprains, while peroneal tendon disorders are comparatively rare but clinically significant sources of chronic lateral ankle pain and instability [1,2,3]. Among these, isolated peroneus longus (PL) tears are particularly uncommon and frequently underdiagnosed [4,5,6].

Surgical repair has historically been advocated for complete ruptures or recalcitrant tears, yet growing evidence supports conservative strategies emphasizing tendon load management, proprioceptive rehabilitation, and biological stimulation techniques [7].

Extracorporeal shockwave therapy (ESWT) has gained attention over the past decade as a non-invasive modality promoting tendon healing and pain modulation. Both radial and focused ESWT (F-ESWT) have shown efficacy in chronic tendinopathies of the Achilles, patellar, and rotator cuff [8,9,10,11,12]. The literature regarding acute tendon injuries remains scarce, where ESWT has also shown promising results in the treatment of Achilles tendon partial tear or other soft tissue injuries [13,14].

The biological effects include increased tenocyte proliferation, collagen synthesis, up-regulation of transforming growth factor-β1 (TGF-β1), vascular endothelial growth factor (VEGF) expression and angiogenesis [15,16,17].

Despite these promising biological effects, evidence supporting the use of ESWT in acute or subacute structural tendon tears remains limited, particularly in comparison with the substantial literature available for chronic tendinopathies. To date, no clinical studies have specifically evaluated the role of ESWT in acute peroneal tendon injuries or isolated peroneus longus tendon tears.

We report a case of a professional football player treated successfully with F-ESWT as part of a multimodal conservative approach, highlighting its clinical, radiological, and functional outcomes and discussing potential mechanisms underlying tendon repair.

## 2. Case Presentation

### 2.1. History and Clinical Examination

A professional male soccer player (central midfielder) in his late twenties presented three days after sustaining a right ankle (dominant leg) injury during a competitive game on synthetic turf. The mechanism involved an inversion and plantarflexion twist of his right ankle, without direct contact with another player. The athlete reported acute lateral and plantar ankle pain with mild swelling and difficulties with weight-bearing. Due to these difficulties, crutches were provided and used for 10 days, with immobilization in ankle cast and full weight-bearing as tolerated. Training was suspended immediately after injury.

The past medical history included several minor ankle sprains, none being severe or leading to a time-loss of play superior to 21 days, with complete recovery and no subjective chronic instability. No other ankle injury, especially no syndesmosis sprain or ankle fracture, was reported.

Physical examination revealed moderate swelling over the lateral ankle, tenderness along the peroneal groove and cuboid region, and pain on resisted plantarflexion and eversion. Hindfoot alignment was varus bilaterally, and was more pronounced on the right ankle. There was no visible hematoma and no peroneal subluxation, and the neurovascular examination was normal.

### 2.2. Imaging

Plain radiographs excluded an osseous injury. Initial ultrasonography at the time of presentation (3 days after injury) revealed thickening and blurred margins of the PL tendon near its plantar course, just distal of the peroneal sulcus of the cuboid, with loss of normal fibrillar pattern. MRI of the right ankle was obtained 4 days after injury and confirmed an isolated 4.1 cm longitudinal tear of the PL tendon with intratendinous and peritendinous fluid on the T2-weighted PDFS coronal sequences (Figure 1). The peroneus brevis tendon and ankle lateral ligaments were intact.

### 2.3. Biomechanical Assessment

Dynamic foot pressure mapping demonstrated lateral forefoot overload, predominantly at the level of the fourth and fifth metatarsophalangeal joints, associated with dynamic hindfoot varus, more marked on the right foot (Figure 2). Gait analysis confirmed increased adduction–supination of the right foot during stance phase (29.6°, vs. 6.8° on the left foot) (Figure 3). Although formal quantitative gait parameters and plantar pressure metrics were not systematically recorded, these biomechanical findings suggested mechanical contributors to peroneus longus tendon overload and helped guide the orthotic correction strategy.

## 3. Treatment Protocol

### 3.1. Conservative Management Protocol

The rehabilitation program integrated three modalities:

Focused Extracorporeal Shockwave Therapy (F-ESWT): Treatment commenced eleven days after injury. While lying supine on the examination table, the lesion site was first determined under ultrasound guidance and then marked. In total, the player received eight sessions using a focused shockwave device (Duolith SD1 T-TOP ultra, STORZ MEDICAL, Tagerwilen, Switzerland). Each session delivered 2500–4000 impulses with a shot frequency of 4 Hz, targeting the plantar portion of the peroneus longus tendon, directly over the tear area, (0–30 mm depth), with an energy flux density (EFD) ranging from 50 to 550 µJ/mm^2^ (total energy 17–36 J per session). Treatment parameters were progressively adjusted based on patient tolerance (VAS ≤ 3 during application), to increase or decrease the EFD. (Table 1).

Physiotherapy: Physiotherapy included edema management, progressive loading, eccentric strengthening of the peroneal and intrinsic foot muscles, proprioceptive training, and supervised football-specific rehabilitation. Rehabilitation progression was based on clinical and functional criteria, including pain-free exercise performance, absence of reactive swelling, restoration of eversion strength compared with the contralateral side, recovery of single-leg balance and hopping capacity, and tolerance of progressive running, cutting, and football-specific drills. Return to competition was authorized only after the completion of full-intensity training sessions without pain or functional limitation under the supervision of the club’s medical staff (see timeline, Figure 4).

Custom Orthotics: Based on the foot mapping pressure and gait analysis, custom foot orthotics with lateral posting (valgus wedge) were prescribed to correct excessive hindfoot varus and redistribute load medially, thus unloading the peroneal tendons. Orthotics were started one month after injury, and prescribed for at least six months, to durably correct the foot position and unload the peroneal tendons.

### 3.2. Clinical Evaluation

Outcomes were measured for pain with the Visual Analog Scale (VAS, 0–10) and function with the American Orthopaedic Foot and Ankle Society (AOFAS) Ankle–Hindfoot Score. Assessments were conducted at baseline, three months, and 18 months post-treatment initiation.

## 4. Results

### 4.1. Clinical Outcomes

The patient reported rapid pain relief after the second F-ESWT session. By six weeks, pain during daily activities was minimal (VAS 1/10), and remained minimal during sports activities at three months. At three months, AOFAS improved from 53 to 95 points, which was maintained at 18 months (Table 2).

Return to sport milestones included training resumption 8 weeks after therapy start and the first competitive match after 12 weeks. No recurrence or secondary symptoms were observed during the 18 months of follow-up.

### 4.2. Radiological Outcomes

A control MRI performed six weeks after therapy initiation showed clear radiological improvement: reduction in peritendinous fluid and reconstitution of the tendon continuity, decreased intratendinous hyperintensity and intratendinous gap, as well as reduction in tear length by ~40% (from 4.1 cm to 2.5 cm). No fatty infiltration or tendon retraction was visible. (Figure 5).

### 4.3. Biomechanical Re-Evaluation

Repeated foot pressure mapping confirmed a correction of lateral column overload with orthotic use and improved gait symmetry, suggesting successful mechanical off-loading (Figure 6).

## 5. Discussion

Isolated PL tendon tears are uncommon, often overshadowed by lateral ligament injuries. Missed diagnoses can lead to chronic pain and tendon degeneration [4,5,6]. MRI remains the gold standard for evaluation, while dynamic ultrasound can guide both diagnosis and therapy targeting.

Shockwaves deliver mechanical energy that stimulates biological cascades promoting tissue regeneration. This direct mechanotransduction induces controlled inflammation and regenerative signaling pathways. Preclinical studies demonstrate increased tenocyte proliferation and collagen synthesis [15], TGF-β1 [16] and VEGF expression, and neovascularization at the tendon–bone junction [17], which may improve local perfusion and accelerate matrix remodeling. Other studies have demonstrated analgesic effects through desensitization of nociceptors, modulating pain through the down-regulation of substance P and calcitonin gene-related peptide [18,19,20].

Recent clinical studies support ESWT’s role in both chronic and acute musculoskeletal conditions [21], including Achilles tendinopathy [22], plantar fasciopathy [23], patellar tendinopathy [24], and calcific shoulder tendinitis [25]. A systematic review and meta-analysis by Elgendy et al. reported significant functional improvement with ESWT across lower-limb tendinopathies [26]. Evidence for ESWT in acute tendon tears, however, remains limited to indirect biological data and isolated reports. Hsu et al. reported the successful treatment of an Achilles tendon partial tear with F-ESWT, with documented tendon healing and pain decrease [13]. Similarly, a systematic review by Zissler et al. showed positive effects of ESWT with improving healing in acute cases, including muscle and tendon injuries [14].

Even though findings from chronic tendinopathies cannot be directly extrapolated to acute injuries, in the present case, the use of F-ESWT, together with a multimodal treatment protocol, was based on a biological rationale supported by preclinical tendon-healing studies and limited clinical evidence in acute soft-tissue injuries and partial tendon tears, rather than on established evidence specific to acute peroneal tendon tears. The favorable evolution observed in the present case may have resulted from the combined effects of early diagnosis, protected progressive rehabilitation, and correction of lateral column overload with custom orthotics, and the potential biological effects induced by F-ESWT. Experimental and clinical studies suggest that ESWT may promote mechanotransduction, neovascularization, modulation of inflammatory mediators, collagen remodeling, and pain reduction. Nevertheless, causality cannot be established from a single case report, and further prospective studies are required to determine the safety, efficacy, and optimal indications of F-ESWT in acute tendon injuries. Therefore, the present case should be interpreted as hypothesis-generating: F-ESWT may represent a useful adjunct within a structured conservative protocol for selected partial acute peroneal tendon tears, but its safety, efficacy, and comparative value require confirmation in larger prospective studies.

Few reports exist on conservative treatment of isolated PL tears, and the available literature mostly concerns chronic injuries or surgical management. Current consensus recommendations for peroneal tendon disorders support individualized management and consideration of conservative treatment in acute cases, while surgical series and systematic reviews have reported positive outcomes when operative repair is required. Dombek et al. described surgical series with favorable outcomes after operative treatment of peroneal tendon tears, while van Dijk et al. emphasized the importance of individualized treatment strategies based on tear pattern, instability, chronicity, and patient demands [4,7]. In high-level athletes, surgical treatment is often considered because of concerns regarding persistent pain, recurrent symptoms, or delayed return to sport, although postoperative rehabilitation commonly requires approximately 4–6 months before full competitive return [6]. To our knowledge, no prior study has documented MRI-confirmed tendon healing after F-ESWT as part of a first-line conservative treatment in an acute isolated peroneus longus tear in a professional athlete.

In the present case, the accelerated return-to-play at 12 weeks after injury compares favorably with surgical rehabilitation (4–6 months) [6], with sustained clinical improvement and no recurrence at the 18-month follow-up. The relatively rapid return-to-play timeline may suggest that the combination of early diagnosis, structured rehabilitation, biomechanical correction, and adjunctive F-ESWT contributed to favorable tendon healing while avoiding surgical morbidity and prolonged postoperative rehabilitation in this specific case. The durability of clinical improvement at 18 months further suggests stable biomechanical and biological restoration.

### 5.1. Limitations

As a single case report, these findings cannot be generalized. Controlled studies comparing F-ESWT with standard conservative or surgical treatments are needed. Additionally, with some missing data regarding F-ESWT (total energy per session or VAS), the results need to be interpreted with caution. The choice of the AOFAS score may also represent a limitation, since it is not purely a patient-reported outcome measure. In the present study, the AOFAS score was, however, selected because of its frequent use in the evaluation of pain and functional recovery after peroneal tendon injuries, allowing comparison with previously published series [27,28]. Another limitation is that the biomechanical assessment was primarily qualitative, as formal quantitative plantar pressure and gait analysis parameters were not systematically recorded. Finally, quantitative biomarkers of healing (e.g., ultrasound elastography, serum cytokine assays) may further help elucidate underlying mechanisms.

### 5.2. Take-Away Lessons

This case underscores several key considerations:

• Early imaging is essential for differentiating peroneal tendon pathology from simple sprains.
• F-ESWT may be initiated early when imaging confirms a partial tear without tendon retraction.
• Integration with physiotherapy and orthotic correction addresses both biological and mechanical healing components.

Given its safety profile and non-invasive nature, F-ESWT could be included in a conservative management protocol to treat select acute tendon tears, especially in high-demand athletes.

## 6. Conclusions

A multimodal treatment protocol including focused extracorporeal shockwave therapy, integrated with physiotherapy, progressive loading and orthotic correction, achieved excellent clinical and MRI outcomes in an isolated peroneus longus tendon tear. This case provides preliminary evidence supporting focused extracorporeal shockwave therapy as a potential adjunct within a multimodal treatment strategy for acute tendon injuries in athletes. The potential role of focused extracorporeal shockwave therapy in acute peroneal tendon injuries requires confirmation through larger prospective and comparative studies.

## Figures and Tables

**Figure 1:**
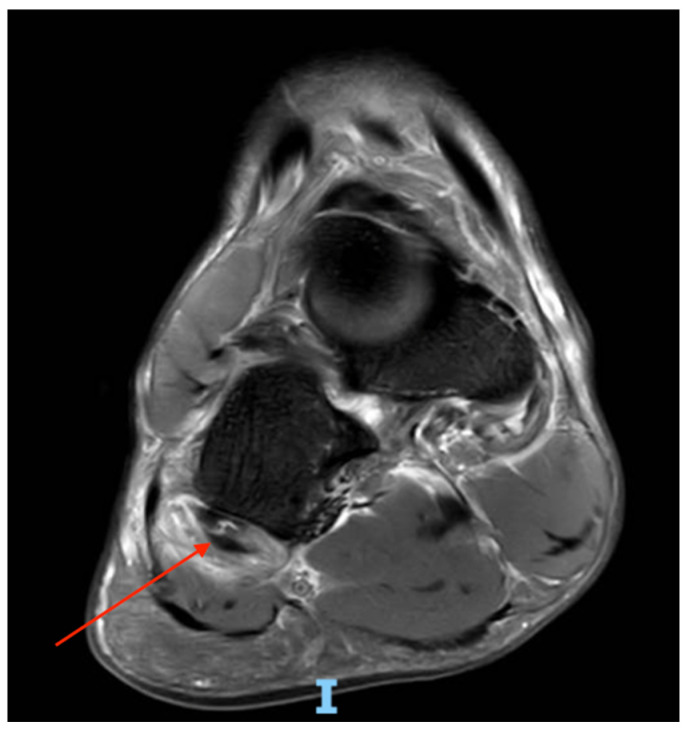
MRI of right ankle (T2 PDFS coronal): longitudinal tear (red arrow) of peroneus longus tendon with intratendinous hyperintensity and fluid.

**Figure 2:**
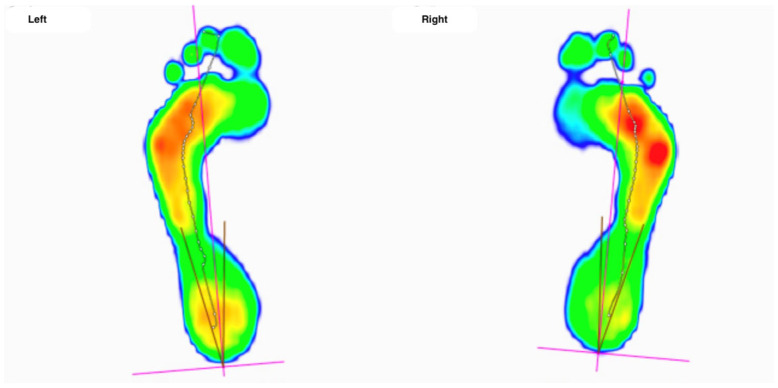
Foot pressure mapping: severe lateral overload at fourth and fifth metatarsophalangeal joints, more pronounced on the right foot. In this qualitative analysis, red indicates the areas of highest pressure, with pressures values progressively decreasing through orange, yellow, and green to blue, which represents the smallest pressure.

**Figure 3:**
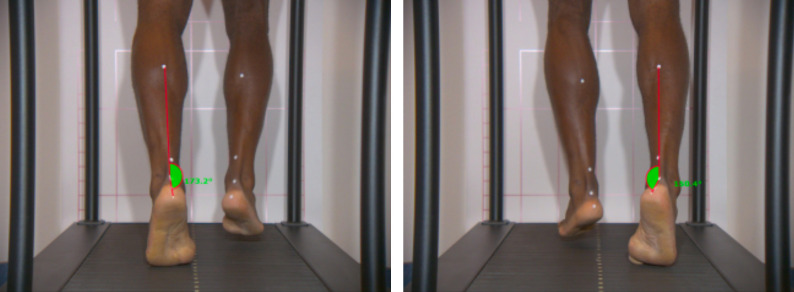
Gait analysis: excessive adduction–supination of the right foot.

**Figure 4:**
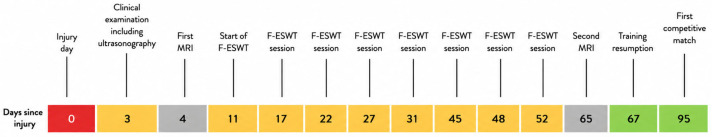
Timeline from injury to return-to-play.

**Figure 5:**
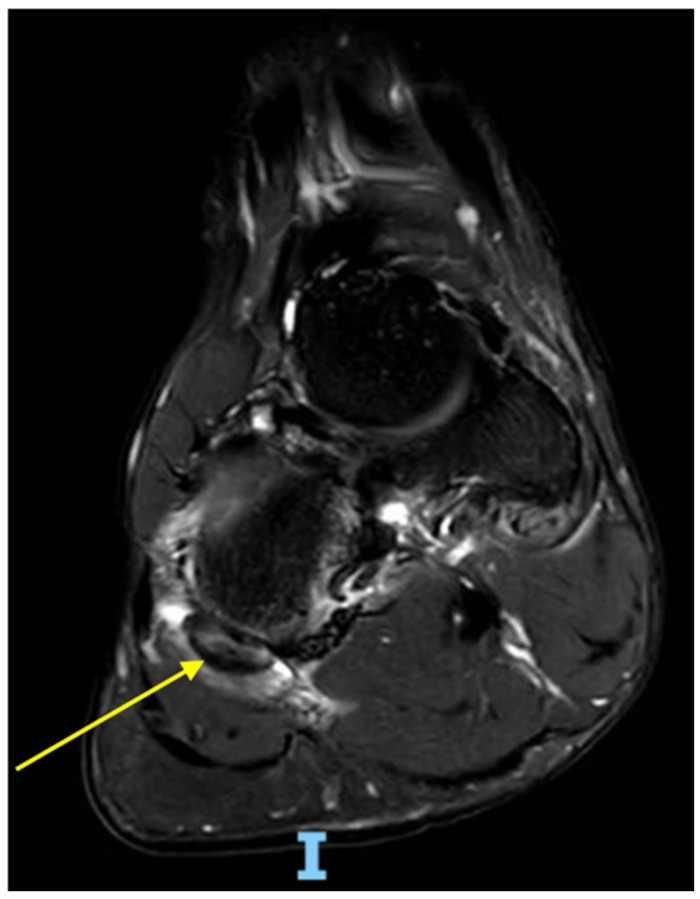
Follow-up MRI (6 weeks): decreased hyperintensity and restored tendon continuity (yellow arrow).

**Figure 6:**
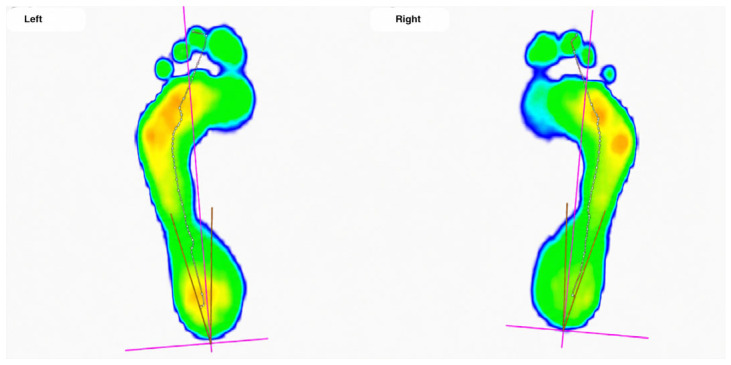
Foot pressure mapping after treatment: decreased lateral overload bilaterally. In this qualitative analysis, red indicates the areas of highest pressure, with pressures values progressively decreasing through orange, yellow, and green to blue, which represents the smallest pressure.

**Table 1:** Focused extracorporeal shockwave therapy parameters per session.

Session Number (Timing Since Injury)	Impulses (Total)	EFD (Total)	VAS Max
1 (D + 11)	4000	50–200 µJ/mm^2^ (20 J)	6/10
2 (D + 17)	2500	50–250 µJ/mm^2^ (17 J)	7/10
3 (D + 22)	3000	50–300 µJ/mm^2^ (24 J)	5/10
4 (D + 27)	3000	100–350 µJ/mm^2^ (24 J)	Missing data
5 (D + 31)	2500	100–500 µJ/mm^2^ (36 J)	8/10
6 (D + 45)	3000	100–500 µJ/mm^2^ (missing data)	6/10
7 (D + 48)	2500	350–550 µJ/mm^2^ (28 J)	4/10
8 (D + 52)	2500	350–550 µJ/mm^2^ (missing data)	3/10

*F-ESWT* Focused extracorporeal shockwave therapy, *D* Day since injury, *EFD* Energy flux density, *J* Joules, *VAS* Visual Analog Scale.

**Table 2:** Clinical outcomes at baseline, 3 months, and last follow-up.

Variable	Time of Injury	3 Months	Last Follow-Up (18 Months)
AOFAS Ankle–Hindfoot Score	53	95	95
Pain	20	40	40
Function	33	45	45
Alignment	5	5	5
VAS	7	1	0

*AOFAS* American Orthopaedic Foot and Ankle Society Score, *VAS* Visual Analog Scale.

## Data Availability

No new data were created or analyzed in this study. Data sharing is not applicable to this article.

## Abbreviations

The following abbreviations are used in this manuscript:

PL	Peroneus longus
F-ESWT	Focused extracorporeal shockwave therapy
VAS	Visual Analog Scale
MRI	Magnetic Resonance Imaging
TGF-β1	Transforming growth factor-β1
VEGF	Vascular endothelial growth factor

## References

[1] López-Valenciano A., Ruiz-Pérez I., Garcia-Gómez A., Vera-Garcia F.J., De Ste Croix M., Myer G.D., Ayala F. (2020). Epidemiology of injuries in professional football: A systematic review and meta-analysis. Br. J. Sports Med..

[2] Peterson L., Junge A., Chomiak J., Graf-Baumann T., Dvorak J. (2000). Incidence of football injuries and complaints in different age groups and skill-level groups. Am. J. Sports Med..

[3] Taljanovic M.S., Alcala J.N., Gimber L.H., Rieke J.D., Chilvers M.M., Latt L.D. (2015). High-resolution US and MR imaging of peroneal tendon injuries. Radiographics.

[4] Dombek M.F., Lamm B.M., Saltrick K., Mendicino R.W., Catanzariti A.R. (2003). Peroneal tendon tears: A retrospective review. J. Foot Ankle Surg..

[5] Peacock K.C., Resnick E.J., Thoder J.J. (1986). Fracture of the os peroneum with rupture of the peroneus longus tendon: A case report and review of the literature. Clin. Orthop. Relat. Res..

[6] van Dijk P.A., Lubberts B., Verheul C., DiGiovanni C.W., Kerkhoffs G.M.M.J. (2016). Rehabilitation after surgical treatment of peroneal tendon tears and ruptures. Knee Surg. Sports Traumatol. Arthrosc..

[7] van Dijk P.A., Miller D., Calder J., DiGiovanni C.W., Kennedy J.G., Kerkhoffs G.M., Kynsburtg A., Havercamp D., Guillo S., Oliva X.M. (2018). The ESSKA-AFAS international consensus statement on peroneal tendon pathologies. Knee Surg. Sports Traumatol. Arthrosc..

[8] Carulli C., Tonelli F., Innocenti M., Gambardella B., Muncibì F., Innocenti M. (2016). Effectiveness of extracorporeal shockwave therapy in three major tendon diseases. J. Orthop. Traumatol..

[9] Xue X., Song Q., Yang X., Kuati A., Fu H., Liu Y., Cui G. (2024). Effect of extracorporeal shockwave therapy for rotator cuff tendinopathy: A systematic review and meta-analysis. BMC Musculoskelet. Disord..

[10] Vetrano M., Castorina A., Vulpiani M.C., Baldini R., Pavan A., Ferretti A. (2013). Platelet-Rich Plasma Versus Focused Shock Waves in the Treatment of Jumper’s Knee in Athletes. Am. J. Sports Med..

[11] Speed C. (2014). A systematic review of shockwave therapies in soft tissue conditions: Focusing on the evidence. Br. J. Sports Med..

[12] Wang C.J. (2012). Extracorporeal shockwave therapy in musculoskeletal disorders. J. Orthop. Surg. Res..

[13] Hsu Y.C., Wu W.T., Chang K.V., Han D.-S., Chou L.-W. (2017). Healing of Achilles tendon partial tear following focused shockwave: A case report and literature review. J. Pain Res..

[14] Zissler A., Stoiber W., Pittner S., Sänger A.M. (2018). Extracorporeal Shock Wave Therapy in Acute Injury Care: A Systematic Review. Rehabil. Process Outcome.

[15] Vetrano M., d’Alessandro F., Torrisi M.R., Ferretti A., Vulpiani M.C., Visco V. (2011). Extracorporeal shock wave therapy promotes cell proliferation and collagen synthesis of primary cultured human tenocytes. Knee Surg. Sports Traumatol. Arthrosc..

[16] Notarnicola A., Moretti B. (2012). The biological effects of extracorporeal shock wave therapy (eswt) on tendon tissue. Muscles Ligaments Tendons J..

[17] Wang C.J., Wang F.S., Yang K.D., Weng L., Hsu C., Huang C., Yang L. (2003). Shock wave therapy induces neovascularization at the tendon-bone junction: A study in rabbits. J. Orthop. Res..

[18] Ryskalin L., Morucci G., Natale G., Soldani P., Gesi M. (2022). Molecular Mechanisms Underlying the Pain-Relieving Effects of Extracorporeal Shock Wave Therapy: A Focus on Fascia Nociceptors. Life.

[19] Müller-Ehrenberg H., Bonavita J., Sun Y., Stecco C., Giordani F. (2025). The State of Extracorporeal Shockwave Therapy for Myofascial Pain Syndrome-A Scoping Review and a Call for Standardized Protocols. Life.

[20] Maier M., Averbeck B., Milz S., Refior H.J., Schmitz C. (2003). Substance P and prostaglandin E2 release after shock wave application to the rabbit femur. Clin. Orthop. Relat. Res..

[21] Simplicio C.L., Purita J., Murrell W., Santos G.S., dos Santos R.G., Lana J.F.S.D. (2020). Extracorporeal shock wave therapy mechanisms in musculoskeletal regenerative medicine. J. Clin. Orthop. Trauma.

[22] Feeney K.M. (2022). The Effectiveness of Extracorporeal Shockwave Therapy for Midportion Achilles Tendinopathy: A Systematic Review. Cureus.

[23] Ogden J.A., Alvarez R., Levitt R., Cross G.L., Marlow M. (2001). Shock wave therapy for chronic proximal plantar fasciitis. Clin. Orthop. Relat. Res..

[24] Mani-Babu S., Morrissey D., Waugh C., Screen H., Barton C. (2015). The effectiveness of extracorporeal shock wave therapy in lower limb tendinopathy: A systematic review. Am. J. Sports Med..

[25] Peters J., Luboldt W., Schwarz W., Jacobi V., Herzog C., Vogl T.J. (2004). Extracorporeal shock wave therapy in calcific tendinitis of the shoulder. Skelet. Radiol..

[26] Elgendy M.H., Khalil S.E., ElMeligie M.M., Elazab D.R. (2024). Effectiveness of extracorporeal shockwave therapy in treatment of upper and lower limb tendinopathies: A systematic review and meta-analysis. Physiother. Res. Int..

[27] Bourgault C., Pougès C., Szymanski C., Lalanne C., Thioun A., Soudy K., Maynou C. (2018). Clinical results of peroneal tendon repair in a retrospective series of 30 patients. Orthop. Traumatol. Surg. Res..

[28] Mercer N.P., Gianakos A.L., Mercurio A.M., Kennedy J.G. (2021). Clinical Outcomes of Peroneal Tendon Tears: A Systematic Review. Foot Ankle Surg..

